# Professor Corneliani on the Proximate Cause and Treatment of Chlorosis

**Published:** 1845-07

**Authors:** 


*Professor Corneliani on the Proximate Cause and Treatment of Chlorosis.*—From an extensive experience in the wards of the Pavia Hospital—to which he is attached as the professor of clinical medicine—the author deduces the following conclusions respecting this not unfrequent disease.

1. The essential nature of chlorosis consists of two pathological conditions, both of them appertaining to the solids:—the *first* being an inordinate excitation of the heart and arteries, and the *second* a chemico-vital alteration of the assimilative functions of Chylification and Hematosis. It is not possible to determine which of these two conditions is the primary and causal one.

2. No plan of treatment is so certain and efficacious as the exhibition of steel in some form or another: the preparations of this metal acting curatively upon both of the pathological states now mentioned.

3. There is no very marked difference in the comparative efficacy of different chalybeate preparations, except in so far as relates to their solubility in the animal fluids, and perhaps also to their readiness to become disaggregated by the process of digestion.

4. The addition of an acid decidedly increases the efficacy of steel remedies.

5. Steel-filings become converted, in the stomach of chlorotic patients, into the lactate of iron.

6. It is useless—and not unfrequently it is unsafe—to administer very large doses of ferruginous preparations.

Professor Corneliani has examined with great care the state of the blood in chlorosis; and most of his observations go to confirm the accuracy of MM. Andral and Gavarret's statements respecting the diminution of the normal proportion of red globules and hematosine.

*Med. Chir. Rev.*

